# New Results on Superlinear Convergence of Classical Quasi-Newton Methods

**DOI:** 10.1007/s10957-020-01805-8

**Published:** 2021-01-09

**Authors:** Anton Rodomanov, Yurii Nesterov

**Affiliations:** 1 ICTEAM, Catholic University of Louvain, Louvain-la-Neuve, Belgium; 2 CORE, Catholic University of Louvain, Louvain-la-Neuve, Belgium

**Keywords:** Quasi-Newton methods, Convex Broyden class, DFP, BFGS, Superlinear convergence, Local convergence, Rate of convergence, 90C53, 90C30, 68Q25

## Abstract

We present a new theoretical analysis of local superlinear convergence of classical quasi-Newton methods from the convex Broyden class. As a result, we obtain a significant improvement in the currently known estimates of the convergence rates for these methods. In particular, we show that the corresponding rate of the Broyden–Fletcher–Goldfarb–Shanno method depends only on the product of the dimensionality of the problem and the *logarithm* of its condition number.

## Introduction

We study local superlinear convergence of classical quasi-Newton methods for smooth unconstrained optimization. These algorithms can be seen as an approximation of the standard Newton method, in which the exact Hessian is replaced by some operator, which is updated in iterations by using the gradients of the objective function. The two most famous examples of quasi-Newton algorithms are the Davidon–Fletcher–Powell (DFP) [1, 2] and the Broyden–Fletcher–Goldfarb–Shanno (BFGS) [3–7] methods, which together belong to the Broyden family [8] of quasi-Newton algorithms. For an introduction into the topic, see [9] and [10, Chapter 6]. See also [11] for the discussion of quasi-Newton algorithms in the context of nonsmooth optimization.

The superlinear convergence of quasi-Newton methods was established as early as in 1970s, firstly by Powell [12] and Dixon [13, 14] for the methods with exact line search, and then by Broyden, Dennis and Moré [15] and Dennis and Moré [16] for the methods without line search. The latter two approaches have been extended onto more general methods under various settings (see, e.g., [17–25]).

However, explicit *rates* of superlinear convergence for quasi-Newton algorithms were obtained only recently. The first results were presented in [26] for the *greedy* quasi-Newton methods. After that, in [27], the *classical* quasi-Newton methods were considered, for which the authors established certain superlinear convergence rates, depending on the problem dimension and its condition number. The analysis was based on the trace potential function, which was then augmented by the logarithm of determinant of the *inverse* Hessian approximation to extend the proof onto the general nonlinear case.

In this paper, we further improve the results of [27]. For the classical quasi-Newton methods, we obtain new convergence rate estimates, which have better dependency on the condition number of the problem. In particular, we show that the superlinear convergence rate of BFGS depends on the condition number only through the *logarithm*. As compared to the previous work, the main difference in the analysis is the choice of the potential function: now the main part is formed by the logarithm of determinant of Hessian approximation, which is then augmented by the trace of *inverse* Hessian approximation.

It is worth noting that recently, in [28], another analysis of local superlinear convergence of the classical DFP and BFGS methods was presented with the resulting rate, which is independent of the dimensionality of the problem and its condition number. However, to obtain such a rate, the authors had to make an additional assumption that the methods start from a sufficiently good initial Hessian approximation. Without this assumption, to our knowledge, their proof technique, based on the Frobenius-norm potential function, leads only to the rates, which are weaker than those in [27].

This paper is organized as follows. In Sect. 2, we introduce our notation. In Sect. 3, we study the convex Broyden class of quasi-Newton updates for approximating a self-adjoint positive definite operator. In Sect. 4, we analyze the rate of convergence of the classical quasi-Newton methods from the convex Broyden class as applied to minimizing a quadratic function. On this simple example, where the Hessian is constant, we illustrate the main ideas of our analysis. In Sect. 5, we consider the general unconstrained optimization problem. Finally, in Sect. 6, we discuss why the new superlinear convergence rates, obtained in this paper, are better than the previously known ones.

## Notation

In what follows, $\mathbb {E}$ denotes an *n*-dimensional real vector space. Its dual space, composed of all linear functionals on $\mathbb {E}$, is denoted by $\mathbb {E}^*$. The value of a linear function $s \in \mathbb {E}^*$, evaluated at a point $x \in \mathbb {E}$, is denoted by $\langle s, x \rangle$.

For a smooth function $f : \mathbb {E}\rightarrow \mathbb {R}$, we denote by $\nabla f(x)$ and $\nabla ^2 f(x)$ its gradient and Hessian, respectively, evaluated at a point $x \in \mathbb {E}$. Note that $\nabla f(x) \in \mathbb {E}^*$, and $\nabla ^2 f(x)$ is a self-adjoint linear operator from $\mathbb {E}$ to $\mathbb {E}^*$.

The partial ordering of self-adjoint linear operators is defined in the standard way. We write $A_1 \preceq A_2$ for $A_1, A_2 : \mathbb {E}\rightarrow \mathbb {E}^*$, if $\langle (A_2 - A_1) x, x \rangle \ge 0$ for all $x \in \mathbb {E}$, and $H_1 \preceq H_2$ for $H_1, H_2 : \mathbb {E}^* \rightarrow \mathbb {E}$, if $\langle s, (H_2 - H_1) s \rangle \ge 0$ for all $s \in \mathbb {E}^*$.

Any self-adjoint positive definite linear operator $A : \mathbb {E}\rightarrow \mathbb {E}^*$ induces in the spaces $\mathbb {E}$ and $\mathbb {E}^*$ the following pair of conjugate Euclidean norms:

1 $$\begin{aligned} \Vert h \Vert _A :=\langle A h, h \rangle ^{1/2}, \quad h \in \mathbb {E}, \qquad \quad \Vert s \Vert _A^* :=\langle s, A^{-1} s \rangle ^{1/2}, \quad s \in \mathbb {E}^*. \end{aligned}$$

When $A = \nabla ^2 f(x)$, where $f : \mathbb {E}\rightarrow \mathbb {R}$ is a smooth function with positive definite Hessian, and $x \in \mathbb {E}$, we prefer to use notation $\Vert \cdot \Vert _x$ and $\Vert \cdot \Vert _x^*$, provided that there is no ambiguity with the reference function *f*.

Sometimes, in the formulas, involving products of linear operators, it is convenient to treat $x \in \mathbb {E}$ as a linear operator from $\mathbb {R}$ to $\mathbb {E}$, defined by $x \alpha = \alpha x$, and $x^*$ as a linear operator from $\mathbb {E}^*$ to $\mathbb {R}$, defined by $x^* s = \langle s, x \rangle$. Likewise, any $s \in \mathbb {E}^*$ can be treated as a linear operator from $\mathbb {R}$ to $\mathbb {E}^*$, defined by $s \alpha = \alpha s$, and $s^*$ as a linear operator from $\mathbb {E}$ to $\mathbb {R}$, defined by $s^* x = \langle s, x \rangle$. In this case, $x x^*$ and $s s^*$ are rank-one self-adjoint linear operators from $\mathbb {E}^*$ to $\mathbb {E}$ and from $\mathbb {E}^*$ to $\mathbb {E}$, respectively, acting as follows: $(x x^*) s = \langle s, x \rangle x$ and $(s s^*) x = \langle s, x \rangle s$ for $x \in \mathbb {E}$ and $s \in \mathbb {E}^*$.

Given two self-adjoint linear operators $A : \mathbb {E}\rightarrow \mathbb {E}^*$ and $H : \mathbb {E}^* \rightarrow \mathbb {E}$, we define the trace and the determinant of *A* with respect to *H* as follows: $\langle H, A \rangle :=\mathrm{Tr}(H A)$, and $\mathrm{Det}(H, A) :=\mathrm{Det}(H A)$. Note that *HA* is a linear operator from $\mathbb {E}$ to itself, and hence, its trace and determinant are well defined by the eigenvalues (they coincide with the trace and determinant of the matrix representation of *HA* with respect to an arbitrary chosen basis in the space $\mathbb {E}$, and the result is independent of the particular choice of the basis). In particular, if *H* is positive definite, then $\langle H, A \rangle$ and $\mathrm{Det}(H, A)$ are, respectively, the sum and the product of the eigenvalues of *A* relative to $H^{-1}$. Observe that $\langle \cdot , \cdot \rangle$ is a bilinear form, and for any $x \in \mathbb {E}$, we have $\langle A x, x \rangle = \langle x x^*, A \rangle$. When *A* is invertible, we also have $\langle A^{-1}, A \rangle = n$ and $\mathrm{Det}(A^{-1}, \delta A) = \delta ^n$ for any $\delta \in \mathbb {R}$. Also recall the following multiplicative formula for the determinant: $\mathrm{Det}(H, A) = \mathrm{Det}(H, G) \cdot \mathrm{Det}(G^{-1}, A)$, which is valid for any invertible linear operator $G : \mathbb {E}\rightarrow \mathbb {E}^*$. If the operator *H* is positive semidefinite, and $A_1 \preceq A_2$ for some self-adjoint linear operators $A_1, A_2 : \mathbb {E}\rightarrow \mathbb {E}^*$, then $\langle H, A_1 \rangle \le \langle H, A_2 \rangle$ and $\mathrm{Det}(H, A_1) \le \mathrm{Det}(H, A_2)$. Similarly, if *A* is positive semidefinite and $H_1 \preceq H_2$ for some self-adjoint linear operators $H_1, H_2 : \mathbb {E}^* \rightarrow \mathbb {E}$, then $\langle H_1, A \rangle \le \langle H_2, A \rangle$ and $\mathrm{Det}(H_1, A) \le \mathrm{Det}(H_2, A)$.

## Convex Broyden Class

Let *A* and *G* be two self-adjoint positive definite linear operators from $\mathbb {E}$ to $\mathbb {E}^*$, where *A* is the target operator, which we want to approximate, and *G* is its current approximation. The *Broyden class* of quasi-Newton updates of *G* with respect to *A* along a direction $u \in \mathbb {E}\setminus \{0\}$ is the following family of updating formulas, parameterized by a scalar $\tau \in \mathbb {R}$:

2 $$\begin{aligned} \begin{array}{rcl} \mathrm{Broyd}_{\tau }(A, G, u) &{}=&{} \phi _{\tau } \left[ G - \frac{A u u^* G + G u u^* A}{\langle A u, u \rangle } + \left( \frac{\langle G u, u \rangle }{\langle A u, u \rangle } + 1 \right) \frac{A u u^* A}{\langle A u, u \rangle } \right] \\ &{}&{} + \, (1 - \phi _{\tau }) \left[ G - \frac{G u u^* G}{\langle G u, u \rangle } + \frac{A u u^* A}{\langle A u, u \rangle } \right] , \end{array} \end{aligned}$$

where

3 $$\begin{aligned} \begin{array}{rcl} \phi _{\tau } \;:=\; \phi _{\tau }(A, G, u):= & {} \frac{\tau \frac{\langle A u, u \rangle }{\langle A G^{-1} A u, u \rangle }}{\tau \frac{\langle A u, u \rangle }{\langle A G^{-1} A u, u \rangle } + (1 - \tau ) \frac{\langle G u, u \rangle }{\langle A u, u \rangle }}. \end{array} \end{aligned}$$

If the denominator in (3) is zero, we left both $\phi _{\tau }$ and $\mathrm{Broyd}_{\tau }(A, G, u)$ undefined. For the sake of convenience, we also set $\mathrm{Broyd}_{\tau } (A, G, u) = G$ for $u = 0$.

In this paper, we are interested in the *convex* Broyden class, which is described by the values of $\tau \in [0, 1]$. Note that for all such $\tau$ the denominator in (3) is always positive for any $u \ne 0$, so both $\phi _{\tau }$ and $\mathrm{Broyd}_{\tau }(A, G, u)$ are well defined; moreover, $\phi _{\tau } \in [0, 1]$. For $\tau = 1$, we have $\phi _{\tau } = 1$, and (2) becomes the DFP update; for $\tau = 0$, we have $\phi _{\tau } = 0$, and (2) becomes the BFGS update.

### Remark 3.1

Usually the Broyden class is defined directly in terms of the parameter $\phi$. However, in the context of this paper, it is more convenient to work with $\tau$ instead of $\phi$. As can be seen from (66), $\tau$ is exactly the weight of the DFP component in the updating formula for the inverse operator.

A basic property of an update from the convex Broyden class is that it preserves the bounds on the eigenvalues with respect to the target operator.

### Lemma 3.1

(see [27, Lemma 2.1]) If $\frac{1}{\xi } A \preceq G \preceq \eta A$ for some $\xi , \eta \ge 1$, then, for any $u \in \mathbb {E}$, and any $\tau \in [0, 1]$, we have $\frac{1}{\xi } A \preceq \mathrm{Broyd}_{\tau }(A, G, u) \preceq \eta A$.

Consider the measure of closeness of *G* to *A* along direction $u \in \mathbb {E}\setminus \{0\}$:

4 $$\begin{aligned} \nu (A, G, u) :=\frac{\langle (G - A) G^{-1} (G - A) u, u \rangle ^{1/2}}{\langle A u, u \rangle ^{1/2}} \;{\mathop {=}\limits ^{(1)}}\; \frac{\Vert (G - A) u \Vert _G^*}{\Vert u \Vert _A}. \end{aligned}$$

Let us present two potential functions, whose improvement after one update from the convex Broyden class can be bounded from below by a certain nonnegative monotonically increasing function of $\nu$, vanishing at zero.

First, consider the *log-det barrier*

5 $$\begin{aligned} V(A, G) = \ln \mathrm{Det}(A^{-1}, G). \end{aligned}$$

It will be useful when $A \preceq G$. Note that in this case $V(A, G) \ge 0$.

### Lemma 3.2

Let $A, G : \mathbb {E}\rightarrow \mathbb {E}^*$ be self-adjoint positive definite linear operators, $A \preceq G \preceq \eta A$ for some $\eta \ge 1$. Then, for any $\tau \in [0, 1]$ and $u \in \mathbb {E}\setminus \{0\}$:

$$\begin{aligned} V(A, G) - V(A, \mathrm{Broyd}_{\tau }(A, G, u)) \ge \ln \left( 1 + ( \tau \frac{1}{\eta } + 1 - \tau ) \nu ^2(A, G, u) \right) . \end{aligned}$$

### Proof

Indeed, denoting $G_+ :=\mathrm{Broyd}_{\tau }(A, G, u)$, we obtain

6 $$\begin{aligned} \begin{array}{rcl} &{}&{} V(A, G) - V(A, G_+) \;{\mathop {=}\limits ^{(5)}}\; \ln \mathrm{Det}(G_+^{-1}, G) \\ &{}{\mathop {=}\limits ^{(67)}}&{} \ln \left( \tau \frac{\langle A u, u \rangle }{\langle A G^{-1} A u, u \rangle } + (1 - \tau ) \frac{\langle G u, u \rangle }{\langle A u, u \rangle } \right) \\ &{}=&{} \ln \left( 1 + \tau \frac{\langle A (A^{-1} - G^{-1}) A u, u \rangle }{\langle A G^{-1} A u, u \rangle } + (1 - \tau ) \frac{\langle (G - A) u, u \rangle }{\langle A u, u \rangle } \right) . \end{array} \end{aligned}$$

Since^1^$0 \preceq G - A \preceq (1 - \frac{1}{\eta }) G$, we have

7 $$\begin{aligned} (G - A) G^{-1} (G - A) \preceq \left( 1 - \frac{1}{\eta } \right) (G - A) \;\preceq \; \frac{1}{1 + \frac{1}{\eta }} (G - A) \;\preceq \; G - A. \end{aligned}$$

Therefore, denoting $\nu :=\nu (A, G, u)$, we can write that

$$\begin{aligned} \frac{\langle (G - A) u, u \rangle }{\langle A u, u \rangle } {\mathop {\ge }\limits ^{(7)}} \frac{\langle (G - A) G^{-1} (G - A) u, u \rangle }{\langle A u, u \rangle } \;{\mathop {=}\limits ^{(4)}}\; \nu ^2, \end{aligned}$$

and, since $A (A^{-1} - G^{-1}) A = G - A - (G - A) G^{-1} (G - A)$, that

$$\begin{aligned} \begin{array}{rcl} \frac{\langle A (A^{-1} - G^{-1}) A u, u \rangle }{\langle A G^{-1} A u, u \rangle } &{}=&{} \frac{\langle (G - A - (G - A) G^{-1} (G - A)) u, u \rangle }{\langle A G^{-1} A u, u \rangle } \;{\mathop {\ge }\limits ^{(7)}}\; \frac{1}{\eta } \frac{\langle (G - A) G^{-1} (G - A) u, u \rangle }{\langle A G^{-1} A u, u \rangle } \\ &{}\ge &{} \frac{1}{\eta } \frac{\langle (G - A) G^{-1} (G - A) u, u \rangle }{\langle A u, u \rangle } \;{\mathop {=}\limits ^{(4)}}\; \frac{1}{\eta } \nu ^2. \end{array} \end{aligned}$$

Substituting the above two inequalities into (6), we obtain the claim.$\square$

Now consider another potential function, the *augmented log-det barrier*:

8 $$\begin{aligned} \psi (G, A) :=\ln \mathrm{Det}(A^{-1}, G) - \langle G^{-1}, G - A \rangle . \end{aligned}$$

As compared to the log-det barrier, this potential function is more universal since it works even if the condition $A \preceq G$ is violated. Note that the augmented log-det barrier is in fact the Bregman divergence, generated by the strictly convex function $d(A) :=-\ln \mathrm{Det}(B^{-1}, A)$, defined on the set of self-adjoint positive definite linear operators from $\mathbb {E}$ to $\mathbb {E}^*$, where $B : \mathbb {E}\rightarrow \mathbb {E}^*$ is an arbitrary fixed self-adjoint positive definite linear operator. Indeed,

9 $$\begin{aligned} \begin{array}{rcl} \psi (G, A) &{}=&{} -\ln \mathrm{Det}(B^{-1}, A) + \ln \mathrm{Det}(B^{-1}, G) - \langle -G^{-1}, A - G \rangle \\ &{}=&{} d(A) - d(G) - \langle \nabla d(G), A - G \rangle \;\ge \; 0. \end{array} \end{aligned}$$

### Remark 3.2

The idea of combining the trace with the logarithm of determinant to form a potential function for the analysis of quasi-Newton methods can be traced back to [29]. Note also that in [27], the authors studied the evolution of $\psi (A, G)$, i.e. the Bregman divergence was centered at *A* instead of *G*.

### Lemma 3.3

For any real $\alpha \ge \beta > 0$, we have $\alpha + \frac{1}{\beta } - 1 \ge 1$, and

10 $$\begin{aligned} \begin{array}{rclrcl} \alpha - \ln \beta - 1\ge & {} \frac{\sqrt{3}}{2 + \sqrt{3}} \ln \left( \alpha + \frac{1}{\beta } - 1 \right)\ge & {} \frac{6}{13} \ln \left( \alpha + \frac{1}{\beta } - 1 \right) . \end{array} \end{aligned}$$

### Proof

We only need to prove the first inequality in (10) since the second one follows from it and the fact that $\frac{\sqrt{3} + 2}{\sqrt{3}} = 1 + \frac{2}{\sqrt{3}} \le 1 + \frac{7}{6} = \frac{13}{6}$ (since $2 \le \frac{7}{2 \sqrt{3}}$).

Let $\beta > 0$ be fixed, and let $\zeta _1 : (1 - \frac{1}{\beta }, +\infty ) \rightarrow \mathbb {R}$ be the function, defined by $\zeta _1(\alpha ) :=\alpha - \frac{\sqrt{3}}{2 + \sqrt{3}} \ln \left( \alpha + \frac{1}{\beta } - 1 \right)$. Note that the domain of $\zeta _1$ includes the point $\alpha = \beta$ since $\beta \ge 2 - \frac{1}{\beta } > 1 - \frac{1}{\beta }$. Let us show that $\zeta _1$ increases on the interval $[\beta , +\infty )$. Indeed, for any $\alpha \ge \beta$, we have

$$\begin{aligned} \begin{array}{rcl} \zeta _1'(\alpha )= & {} 1 - \frac{\sqrt{3}}{2 + \sqrt{3}} \frac{1}{\alpha + \frac{1}{\beta } - 1} \;>\; 1 - \frac{1}{\alpha + \frac{1}{\beta } - 1} \;=\; \frac{\alpha + \frac{1}{\beta } - 2}{\alpha + \frac{1}{\beta } - 1} \;\ge \; \frac{\beta + \frac{1}{\beta } - 2}{\alpha + \frac{1}{\beta } - 1} \;\ge \; 0. \end{array} \end{aligned}$$

Thus, it is sufficient to prove (10) only in the case when $\alpha = \beta$. Equivalently, we need to show that the function $\zeta _2 : (0, +\infty ) \rightarrow \mathbb {R}$, defined by the formula $\zeta _2 (\alpha ) :=\alpha - \ln \alpha - 1 - \frac{ \sqrt{3}}{2 + \sqrt{3}} \ln \left( \alpha + \frac{1}{\alpha } - 1 \right)$, is nonnegative. Differentiating, we find that, for all $\alpha > 0$, we have

$$\begin{aligned} \begin{array}{rcl} \zeta _2'(\alpha ) &{}=&{} 1 - \frac{1}{\alpha } - \frac{\sqrt{3}}{2 + \sqrt{3}} \frac{1 - \frac{1}{\alpha ^2}}{\alpha + \frac{1}{\alpha } - 1} \;=\; \left( 1 - \frac{1}{\alpha } \right) \left( 1 - \frac{\sqrt{3}}{2 + \sqrt{3}} \frac{1 + \frac{1}{\alpha }}{\alpha + \frac{1}{\alpha } - 1} \right) \\ &{}=&{} \left( 1 - \frac{1}{\alpha } \right) \frac{\alpha + \frac{1}{\alpha } - 1 - (2 \sqrt{3} - 3) (1 + \frac{1}{\alpha })}{\alpha + \frac{1}{\alpha } - 1} \;=\; \left( 1 - \frac{1}{\alpha } \right) \frac{\alpha - 2 (\sqrt{3} - 1) + (\sqrt{3} - 1)^2 \frac{1}{\alpha }}{1 + \frac{1}{\alpha } - 1} \\ &{}=&{} \left( 1 - \frac{1}{\alpha } \right) \frac{( \sqrt{\alpha } - (\sqrt{3} - 1) \frac{1}{\sqrt{\alpha }})^2}{\alpha + \frac{1}{\alpha } - 1}. \end{array} \end{aligned}$$

Hence, $\zeta _2'(\alpha ) \le 0$ for $0 < \alpha \le 1$, and $\zeta _2'(\alpha ) \ge 0$ for $\alpha \ge 1$. Thus, the minimum of $\zeta _2$ is attained at $\alpha = 1$. Consequently, $\zeta _2(\alpha ) \ge \zeta _2(1) = 0$ for all $\alpha > 0$.$\square$

It turns out that, up to some constants, the improvement in the augmented log-det barrier can be bounded from below by exactly the same logarithmic function of $\nu$, which was used for the simple log-det barrier.

### Lemma 3.4

Let $A, G : \mathbb {E}\rightarrow \mathbb {E}^*$ be self-adjoint positive definite linear operators, $\frac{1}{\xi } A \preceq G \preceq \eta A$ for some $\xi , \eta \ge 1$. Then, for any $\tau \in [0, 1]$ and $u \in \mathbb {E}\setminus \{0\}$:

$$\begin{aligned} \psi (G, A) - \psi (\mathrm{Broyd}_{\tau }(A, G, u), A) \ge \frac{6}{13} \ln \left( 1 + ( \tau \frac{1}{\xi \eta } + 1 - \tau ) \nu ^2(A, G, u) \right) . \end{aligned}$$

### Proof

Indeed, denoting $G_+ :=\mathrm{Broyd}_{\tau }(A, G, u)$, we obtain

$$\begin{aligned} \begin{array}{rcl} \langle G^{-1} - G_+^{-1}, A \rangle&{\mathop {=}\limits ^{(66)}}&\tau \left[ \frac{\langle A G^{-1} A G^{-1} A u, u \rangle }{\langle A G^{-1} A u, u \rangle } - 1 \right] + (1 - \tau ) \left[ \frac{\langle A G^{-1} A u, u \rangle }{\langle A u, u \rangle } - 1 \right] , \end{array} \end{aligned}$$

and

$$\begin{aligned} \mathrm{Det}(G_+^{-1}, G) {\mathop {=}\limits ^{(67)}} \tau \frac{\langle A u, u \rangle }{\langle A G^{-1} A u, u \rangle } + (1 - \tau ) \frac{\langle A u, u \rangle }{\langle G u, u \rangle }. \end{aligned}$$

Thus,

11 $$\begin{aligned} \begin{array}{rcl} &{}&{} \psi (G, A) - \psi (G_+, A) \;{\mathop {=}\limits ^{(8)}}\; \langle G^{-1} - G_+^{-1}, A \rangle + \ln \mathrm{Det}(G_+^{-1}, G) \\ &{}&{} \;=\; \tau \alpha _1 + (1 - \tau ) \alpha _0 + \ln (\tau \beta _1^ {-1} + (1 - \tau ) \beta _0^{-1} ) - 1 \;=\; \alpha - \ln \beta - 1, \end{array} \end{aligned}$$

where we denote $\alpha _1 :=\frac{\langle A G^{-1} A G^ {-1} A u, u \rangle }{\langle A G^{-1} A u, u \rangle }$, $\beta _1 :=\frac{\langle A G^{-1} A u, u \rangle }{\langle A u, u \rangle }$, $\alpha _0 :=\frac{\langle A G^{-1} A u, u \rangle }{\langle A u, u \rangle }$, $\beta _0 :=\frac{\langle A u, u \rangle }{\langle G u, u \rangle }$, $\alpha :=\tau \alpha _1 + (1 - \tau ) \alpha _0$, $\beta :=( \tau \beta _1^{-1} + (1 - \tau ) \beta _0^{-1} )^{-1}$. Note that $\alpha _1 \ge \beta _1$ and $\alpha _0 \ge \beta _0$ by the Cauchy–Schwartz inequality. At the same time, $\tau \beta _1 + (1 - \tau ) \beta _2 \ge \beta$ by the convexity of the inverse function $t \mapsto t^{-1}$. Hence, we can apply Lemma 3.3 to estimate (11) from below. Note that

$$\begin{aligned}{}\begin{array}[b]{rcl} \alpha + \frac{1}{\beta } - 1 &{}=&{} \tau \frac{\langle (A + A G^{-1} A G^{-1} A) u, u \rangle }{\langle A G^ {-1} A u, u \rangle } + (1 - \tau ) \frac{\langle (G + A) u, u \rangle }{\langle A u, u \rangle } - 1 \\ &{}=&{} 1 + \tau \frac{\langle (G - A) G^{-1} A G^{-1} (G - A) \rangle }{\langle A G^{-1} A u, u \rangle } + (1 - \tau ) \frac{\langle (G - A) G^ {-1} (G - A) u, u \rangle }{\langle A u, u \rangle } \\ &{}\ge &{} 1 + (\tau \frac{1}{\xi \eta } + 1 - \tau ) \frac{\langle (G - A) G^{-1} (G - A) u, u \rangle }{\langle A u, u \rangle } \\ &{}{\mathop {=}\limits ^{(4)}}&{} 1 + (\tau \frac{1}{\xi \eta } + 1 - \tau ) \nu ^2(A, G, u). \end{array}\quad \quad \square \end{aligned}$$

The measure $\nu (A, G, u)$, defined in (4), is the ratio of the norm of $(G - A) u$, measured with respect to *G*, and the norm of *u*, measured with respect to *A*. It is important that we can change the corresponding metrics to $G_+$ and *G*, respectively, by paying only with the minimal eigenvalue of *G* relative to *A*.

### Lemma 3.5

Let $A, G : \mathbb {E}\rightarrow \mathbb {E}^*$ be self-adjoint positive definite linear operators such that $\frac{1}{\xi } A \preceq G$ for some $\xi > 0$. Then, for any $\tau \in [0, 1]$, any $u \in \mathbb {E}\setminus \{0\}$, and $G_+ :=\mathrm{Broyd}_{\tau }(A, G, u)$, we have

$$\begin{aligned} \nu ^2(A, G, u) \ge \frac{1}{1 + \xi } \frac{\langle (G - A) G_+^{-1} (G - A) u, u \rangle }{\langle G u, u \rangle }. \end{aligned}$$

### Proof

From (66), it is easy to see that $G_+^{-1} A u = u$. Hence,

12 $$\begin{aligned} \begin{array}{rcl} \frac{\langle (G - A) G_+^{-1} (G - A) u, u \rangle }{\langle G u, u \rangle } &{}=&{} \frac{\langle G G_+^{-1} G u, u \rangle }{\langle G u, u \rangle } + \frac{\langle A u, G_+^{-1} A u \rangle }{\langle G u, u \rangle } - 2 \frac{\langle G u, G_+^{-1} A u \rangle }{\langle G u, u \rangle } \\ &{}=&{} \frac{\langle G G_+^{-1} G u, u \rangle }{\langle G u, u \rangle } + \frac{\langle A u, u \rangle }{\langle G u, u \rangle } - 2. \end{array} \end{aligned}$$

Since $1 - t \le \frac{1}{t} - 1$ for all $t > 0$, we further have

13 $$\begin{aligned} \begin{array}{rcl} \frac{\langle G G_+^{-1} G u, u \rangle }{\langle G u, u \rangle } &{}{\mathop {=}\limits ^{(66)}}&{} \tau \left[ 1 - \frac{\langle A u, u \rangle ^2}{\langle G u, u \rangle \langle A G^{-1} A u, u \rangle } + \frac{\langle G u, u \rangle }{\langle A u, u \rangle } \right] \\ &{}&{} + \, (1 - \tau ) \left[ \left( \frac{\langle A G^{-1} A u, u \rangle }{\langle A u, u \rangle } + 1 \right) \frac{\langle G u, u \rangle }{\langle A u, u \rangle } - 1 \right] \\ &{}\le &{} \left( \frac{\langle A G^{-1} A u, u \rangle }{\langle A u, u \rangle } + 1 \right) \frac{\langle G u, u \rangle }{\langle A u, u \rangle } - 1. \end{array} \end{aligned}$$

Denote $$\nu :=\nu (A, G, u)$$. Then,

14 $$\begin{aligned} \begin{array}{rcl} \nu ^2&{\mathop {=}\limits ^{(4)}}&\frac{\langle (G - A) G^{-1} (G - A) u, u \rangle }{\langle A u, u \rangle } \;=\; \frac{\langle G u, u \rangle }{\langle A u, u \rangle } + \frac{\langle A G^{-1} A u, u \rangle }{\langle A u, u \rangle } - 2. \end{array} \end{aligned}$$

Consequently,

15 $$\begin{aligned} \begin{array}{rcl}  (1 + \xi ) \nu ^2 &{}\ge &{} \left( \frac{\langle A G^{-1} A u, u \rangle }{\langle A u, u \rangle } + 1 \right) \nu ^2 \\ &{}{\mathop {=}\limits ^{(14)}}&{} \left( \frac{\langle A G^{-1} A u, u \rangle }{\langle A u, u \rangle } + 1 \right) \frac{\langle G u, u \rangle }{\langle A u, u \rangle } + \frac{\langle A G^{-1} A u, u \rangle ^2}{\langle A u, u \rangle ^2} - \frac{\langle A G^{-1} A u, u \rangle }{\langle A u, u \rangle } - 2 \\ &{}{\mathop {\ge }\limits ^{(13)}}&{} \frac{\langle G G_+^{-1} G u, u \rangle }{\langle A u, u \rangle } + \frac{\langle A G^{-1} A u, u \rangle ^2}{\langle A u, u \rangle } - \frac{\langle A G^{-1} A u, u \rangle }{\langle A u, u \rangle } - 1. \end{array} \end{aligned}$$

Thus,

$$\begin{aligned} \begin{array}{rcl} (1 + \xi ) \nu ^2 - \frac{\langle (G - A) G_+^{-1} (G - A) u, u \rangle }{\langle G u, u \rangle } &{}{\mathop {=}\limits ^{(12)}}&{} (1 + \xi ) \nu ^2 - \frac{\langle G G_+^{-1} G u, u \rangle }{G u, u \rangle } - \frac{\langle A u, u \rangle }{\langle G u, u \rangle } + 2 \\ &{}{\mathop {\ge }\limits ^{(15)}}&{} \frac{\langle A G^{-1} A u, u \rangle ^2}{\langle A u, u \rangle ^2} - \frac{\langle A G^{-1} A u, u \rangle }{\langle A u, u \rangle } - \frac{\langle A u, u \rangle }{\langle G u, u \rangle } + 1 \\ &{}\ge &{} \frac{\langle A G^{-1} A u, u \rangle ^2}{\langle A u, u \rangle ^2} - 2 \frac{\langle A G^{-1} A u, u \rangle }{\langle A u, u \rangle } + 1 \;\ge \; 0, \end{array} \end{aligned}$$

where we have used the Cauchy–Schwartz inequality $\frac{\langle A u, u \rangle }{\langle G u, u \rangle } \le \frac{\langle A G^ {-1} A u, u \rangle }{\langle A u, u \rangle }$.$\square$

## Unconstrained Quadratic Minimization

Let us study the convergence properties of the classical quasi-Newton methods from the convex Broyden class, as applied to minimizing the quadratic function

16 $$\begin{aligned} f(x) :=\frac{1}{2} \langle A x, x \rangle - \langle b, x \rangle , \end{aligned}$$

where $A : \mathbb {E}\rightarrow \mathbb {E}^*$ is a self-adjoint positive definite linear operator, and $b \in \mathbb {E}^*$.

Let $B : \mathbb {E}\rightarrow \mathbb {E}^*$ be a fixed self-adjoint positive definite linear operator, and let $\mu , L > 0$ be such that

17 $$\begin{aligned} \mu B \preceq A \preceq L B. \end{aligned}$$

Thus, $\mu$ is the *strong convexity* parameter of *f*, and *L* is the constant of *Lipschitz continuity* of the gradient of *f*, both measured relative to *B*.

Consider the following standard quasi-Newton process for minimizing (16):
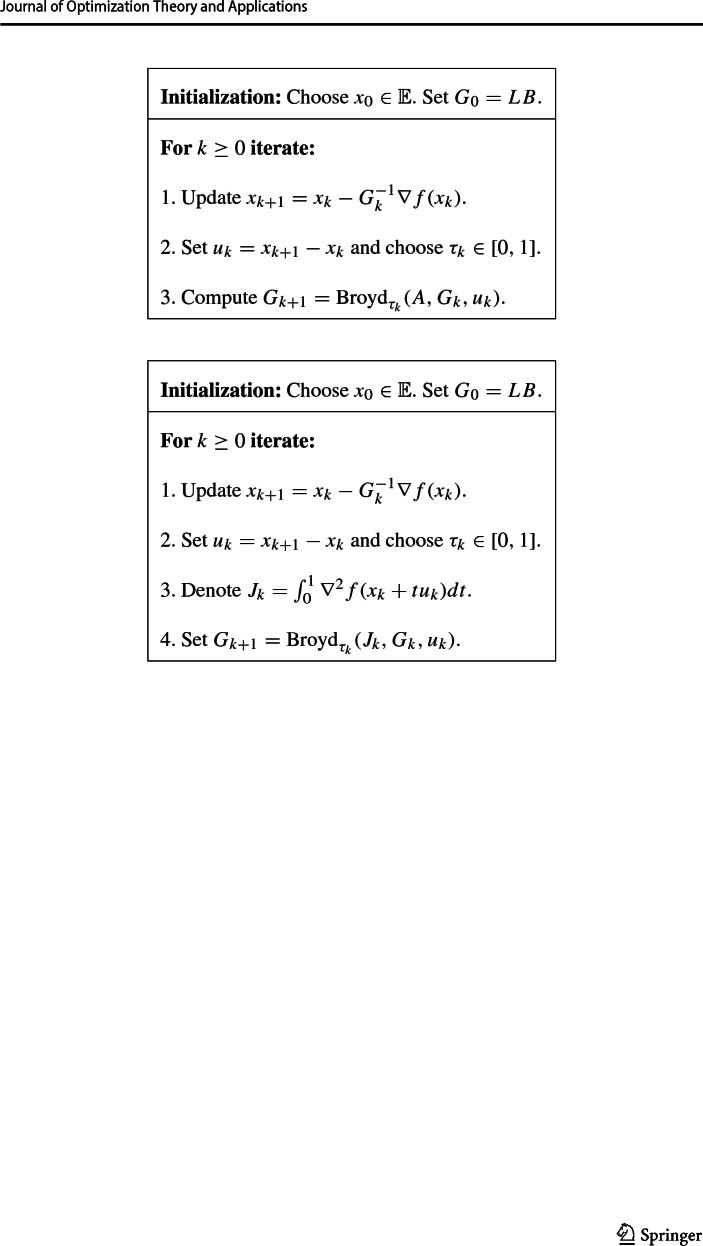
 For measuring its rate of convergence, we use the norm of the gradient, taken with respect to the Hessian:

$$\begin{aligned} \lambda _k :=\Vert \nabla f(x_k) \Vert _A^* \;{\mathop {=}\limits ^{(1)}}\; \langle \nabla f(x_k), A^{-1} \nabla f(x_k) \rangle ^ {1/2}. \end{aligned}$$

It is known that the process () has at least a linear convergence rate of the standard gradient method:

### Theorem 4.1

(see [27, Theorem 3.1]) In scheme (), for all $k \ge 0$:

19 $$\begin{aligned} A \preceq G_k \preceq \frac{L}{\mu } A, \qquad \lambda _k \le \left( 1 - \frac{\mu }{L} \right) ^k \, \lambda _0. \end{aligned}$$

Let us establish the superlinear convergence. According to (19), for the quadratic function, we have $A \preceq G_k$ for all $k \ge 0$. Therefore, in our analysis, we can use both potential functions: the log-det barrier and the augmented log-det barrier. Let us consider both options. We start with the first one.

### Theorem 4.2

In scheme (), for all $k \ge 1$, we have

20 $$\begin{aligned} \lambda _k \le \left[ \frac{2}{\prod _{i=0}^{k-1} (\tau _i \frac{\mu }{L} + 1 - \tau _i)^{1/k}} \left( e^{\frac{n}{k} \ln \frac{L}{\mu }} - 1 \right) \right] ^{k/2} \sqrt{\frac{L}{\mu }} \cdot \lambda _0. \end{aligned}$$

### Proof

Without loss of generality, we can assume that $u_i \ne 0$ for all $0 \le i \le k$. Denote $V_i :=V(A, G_i)$, $\nu _i :=\nu (A, G_i, u_i)$, $p_i :=\tau _i \frac{\mu }{L} + 1 - \tau _i$, $g_i :=\Vert \nabla f(x_i) \Vert _ {G_i}^*$ for any $0 \le i \le k$. By Lemma 3.2 and (19), for all $0 \le i \le k - 1$, we have $\ln ( 1 + p_i \nu _i^2 ) \le V_i - V_ {i+1}$. Summing up, we obtain

21 $$\begin{aligned} \begin{array}{rcl} &{}&{}\sum \limits _{i=0}^{k-1} \ln (1 + p_k \nu _k^2) \;\le \; V_0 - V_k \;{\mathop {\le }\limits ^{(19)}}\; V_0 \;{\mathop {=}\limits ^{(18)}}\; V(A, L B) \\ &{}{\mathop {=}\limits ^{(5)}}&{} \ln \mathrm{Det}(A^{-1}, L B) \;{\mathop {\le }\limits ^{(17)}}\; \ln \mathrm{Det}(\frac{1}{\mu } B^{-1}, L B) \;=\; n \ln \frac{L}{\mu }. \end{array} \end{aligned}$$

Hence, by the convexity of function $t \mapsto \ln (1 + e^t)$, we get

22 $$\begin{aligned} \begin{array}{rcl} \frac{n}{k} \ln \frac{L}{\mu } &{}{\mathop {\ge }\limits ^{(21)}}&{} \frac{1}{k} \sum \limits _{i=0}^{k-1} \ln (1 + p_i \nu _i^2) \;=\; \frac{1}{k} \sum \limits _{i=0}^{k-1} \ln (1 + e^{\ln (p_i \nu _i^2)}) \\ &{}\ge &{} \ln \left( 1 + e^{\frac{1}{k} \sum _{i=0}^{k-1} \ln (p_i \nu _i^2)} \right) \;=\; \ln \left( 1 + \left[ \prod \limits _{i=0}^{k-1} p_i \nu _i^2 \right] ^{1/k} \right) . \end{array} \end{aligned}$$

But, for all $0 \le i \le k - 1$, we have $\nu _i^2 \ge \frac{1}{2} \frac{\langle (G_i - A) G_{i+1}^{-1} (G_i - A) u_i, u_i \rangle }{\langle G_i u_i, u_i \rangle } = \frac{1}{2} \frac{g_ {i+1}^2}{g_i^2}$ by Lemma 3.5, (19), and since $G_i u_i = -\nabla f (x_i)$, $A u_i = \nabla f(x_{i+1}) - \nabla f(x_i)$. Hence, $\prod _ {i=0}^ {k-1} \nu _i^2 \ge \frac{1}{2^k} \frac{g_k^2}{g_0^2}$, and so $\frac{n}{k} \ln \frac{L}{\mu } {\mathop {\ge }\limits ^{(22)}} \ln \left( 1 + \frac{1}{2} \left[ \prod _{i=0}^{k-1} p_i \right] ^{1/k} \left[ \frac{g_k}{g_0} \right] ^{2/k} \right)$. Rearranging, we obtain $g_k \le \left[ \frac{2}{\prod _{i=0}^{k-1} p_i^{1/k}} ( e^{\frac{n}{k} \ln \frac{L}{\mu }} - 1 ) \right] ^{k/2} g_0$. It remains to note that $\lambda _k \le \sqrt{\frac{L}{\mu }} \cdot g_k$ and $g_0 \le \lambda _0$ in view of (19).$\square$

### Remark 4.1

As can be seen from (21), the factor $n \ln \frac{L}{\mu }$ in (20) can be improved up to $\ln \mathrm{Det}(A^{-1}, L B) = \sum _{i=1}^n \ln \frac{L}{\lambda _i}$, where $\lambda _1, \dots , \lambda _n$ are the eigenvalues of *A* relative to *B*. This improved factor can be significantly smaller than the original one if the majority of the eigenvalues $\lambda _i$ are much larger than $\mu$.

Let us briefly present another approach, which is based on the *augmented* log-det barrier. The resulting efficiency estimate will be the same as in Theorem 4.2 up to a slightly worse absolute constant under the exponent. However, this proof can be extended onto general nonlinear functions.

### Theorem 4.3

In scheme (), for all $k \ge 1$, we have

$$\begin{aligned} \lambda _k \le \left[ \frac{2}{\prod _{i=0}^{k-1} (\tau _i \frac{\mu }{L} + 1 - \tau _i)^{1/k}} \left( e^{ \frac{13}{6} \frac{n}{k} \ln \frac{L}{\mu }} - 1 \right) \right] ^{k/2} \sqrt{\frac{L}{\mu }} \cdot \lambda _0. \end{aligned}$$

### Proof

Without loss of generality, we can assume that $u_i \ne 0$ for all $0 \le i \le k$. Denote $\psi _i :=\psi (G_i, A)$, $\nu _i :=\nu (A, G_i, u_i)$, $p_i = \tau _i \frac{\mu }{L} + 1 - \tau _i$, $g_i :=\Vert \nabla f(x_i) \Vert _{G_i}^*$ for all $0 \le i \le k$. By Lemma 3.4 and (19), for all $0 \le i \le k - 1$, we have $\frac{6}{13} \ln (1 + p_i \nu _i^2) \le \psi _i - \psi _{i+1}$. Hence,

23 $$\begin{aligned} \begin{array}{rcl} \frac{6}{13} \sum \limits _{i=0}^{k-1} \ln (1 + p_i \nu _i^2) &{}\le &{} \psi _0 - \psi _k \;{\mathop {\le }\limits ^{(9)}}\; \psi _0 \;{\mathop {=}\limits ^{(18)}}\; \psi (L B, A) \\ &{}{\mathop {=}\limits ^{(8)}}&{} \ln \mathrm{Det}(A^{-1}, L B) - \langle \frac{1}{L} B^ {-1}, L B - A \rangle \;{\mathop {\le }\limits ^{(17)}}\; n \ln \frac{L}{\mu }, \end{array} \end{aligned}$$

and we can continue exactly as in the proof of Theorem 4.2.$\square$

## Minimization of General Functions

In this section, we consider the general unconstrained minimization problem:

24 $$\begin{aligned} \begin{array}{rcl} \min \limits _{x \in \mathbb {E}} f(x), \end{array} \end{aligned}$$

where $f : \mathbb {E}\rightarrow \mathbb {R}$ is a twice continuously differentiable function with positive definite second derivative. Our goal is to study the convergence properties of the following standard quasi-Newton scheme for solving (24): 
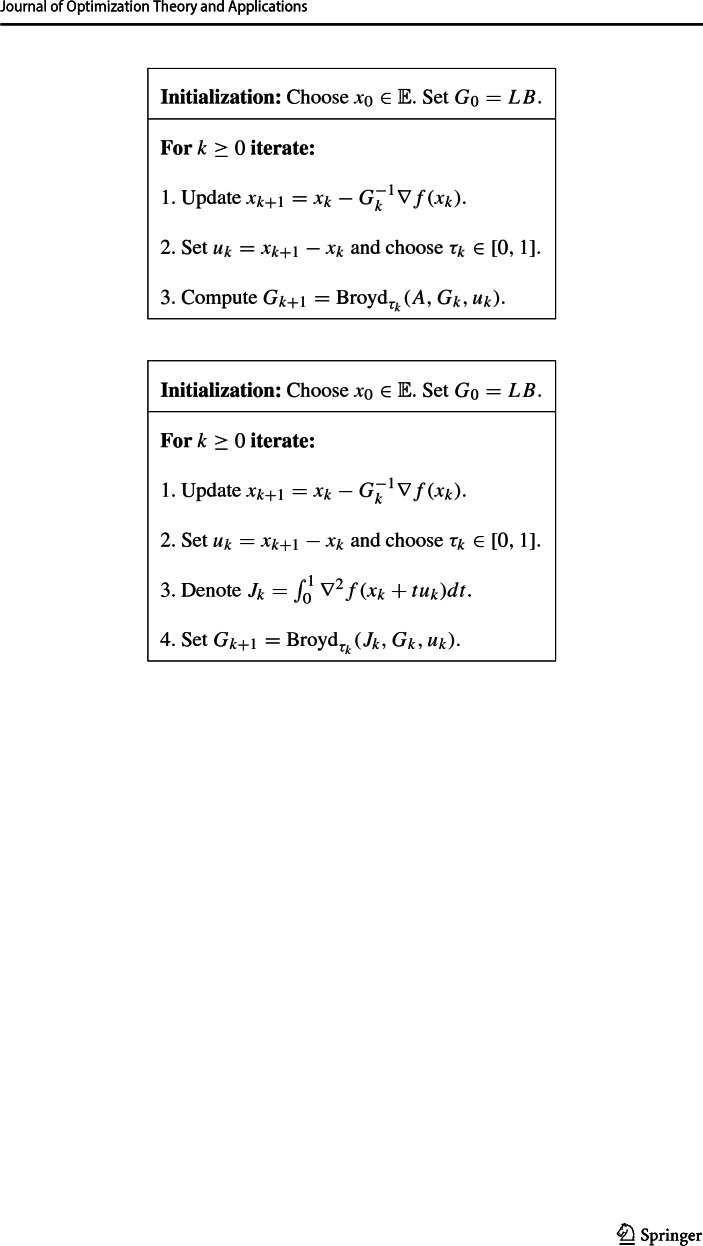
 Here, $B : \mathbb {E}\rightarrow \mathbb {E}^*$ is a self-adjoint positive definite linear operator, and *L* is a positive constant, which together define the initial Hessian approximation $G_0$.

We assume that there exist constants $\mu > 0$ and $M \ge 0$, such that

26 $$\begin{aligned}&\mu B \preceq \nabla ^2 f(x)\preceq L B, \end{aligned}$$

27 $$\begin{aligned}&\nabla ^2 f(y) - \nabla ^2 f(x) \preceq M \Vert y - x \Vert _z \nabla ^2 f(w) \end{aligned}$$

for all $x, y, z, w \in \mathbb {E}$. The first assumption (26) specifies that, relative to the operator *B*, the objective function *f* is $\mu$-*strongly convex* and its gradient is *L*-*Lipschitz continuous*. The second assumption (27) means that *f* is *M*-*strongly self-concordant*. This assumption was recently introduced in [26] as a convenient affine-invariant alternative to the standard assumption of the Lipschitz second derivative and is satisfied at least for any strongly convex function with Lipschitz continuous Hessian (see [26, Example 4.1]). The main facts, which we use about strongly self-concordant functions, are summarized in the following lemma (see [26, Lemma 4.1]):

### Lemma 5.1

For any $$x, y \in \mathbb {E}$$, $$J :=\int _0^1 \nabla ^2 f(x + t (y - x)) dt$$, $$r :=\Vert y - x \Vert _x$$:

28 $$\begin{aligned}&\left( 1 + \frac{M r}{2} \right) ^{-1} \nabla ^2 f(x) \preceq J \preceq \left( 1 + \frac{M r}{2} \right) \nabla ^2 f(x),\end{aligned}$$

29 $$\begin{aligned}&\left( 1 + \frac{M r}{2} \right) ^{-1} \nabla ^2 f(y) \preceq J \preceq \left( 1 + \frac{M r}{2} \right) \nabla ^2 f(y). \end{aligned}$$

Note that for a quadratic function, we have $M = 0$.

For measuring the convergence rate of (), we use the local gradient norm:

30 $$\begin{aligned} \lambda _k :=\Vert \nabla f(x_k) \Vert _{x_k}^* \;{\mathop {=}\limits ^{(1)}}\; \langle \nabla f(x_k), \nabla ^2 f(x_k)^{-1} \nabla f(x_k) \rangle ^{1/2}. \end{aligned}$$

The local convergence analysis of the scheme () is, in general, the same as the corresponding analysis in the quadratic case. However, it is much more technical due to the fact that, in the nonlinear case, the Hessian is no longer constant. This causes a few problems.

First, there are now several different ways how one can treat the Hessian approximation $G_k$. One can view it as an approximation to the Hessian $\nabla ^2 f(x_k)$ at the current iterate $x_k$, to the Hessian $\nabla ^2 f(x^*)$ at the minimizer $x^*$, to the integral Hessian $J_k$, etc. Of course, locally, due to strong self-concordancy, all these variants are equivalent since the corresponding Hessians are close to each other. Nevertheless, from the viewpoint of technical simplicity of the analysis, some options are slightly more preferable than others. We find it to be the most convenient to always think of $G_k$ as an approximation to the integral Hessian $J_k$.

The second issue is as follows. Suppose we already know what is the connection between our current Hessian approximation $G_k$ and the actual integral Hessian $J_k$, e.g., in terms of the relative eigenvalues and the value of the augmented log-det barrier potential function (8). Naturally, we want to know how these quantities change after we update $G_k$ into $G_{k+1}$ at Step 4 of the scheme (). For this, we apply Lemma 3.1 and Lemma 3.4, respectively. However, the problem is that both of these lemmas will provide us only with the information on the connection between the update result $G_{k+1}$ and the *current* integral Hessian $J_k$ (which was used for performing the update), not the next one $J_{k+1}$. Therefore, we need to additionally take into account the errors, resulting from approximating $J_{k+1}$ by $J_k$.

For estimating the errors, which accumulate as a result of approximating one Hessian by another, it is convenient to introduce the following quantities^2^:

31 $$\begin{aligned} r_k :=\Vert u_k \Vert _{x_k}, \qquad \xi _k :=e^{M \sum _{i=0}^{k-1} r_i} \quad (\;\ge \; 1), \qquad k \ge 0. \end{aligned}$$

### Remark 5.1

The general framework of our analysis is the same as in the previous paper [27]. The main difference is that now another potential function is used for establishing the rate of superlinear convergence (Lemma 5.4). However, in order to properly incorporate the new potential function into the analysis, many parts in the proof had to be appropriately modified, most notably the part, related to estimating the region of local convergence. In any case, the analysis, presented below, is fully self-contained and does not require the reader first go through [27].

We analyze the method () in several steps. The first step is to establish the bounds on the relative eigenvalues of the Hessian approximations with respect to the corresponding Hessians.

### Lemma 5.2

For all $k \ge 0$, we have

32 $$\begin{aligned}&\frac{1}{\xi _k} \nabla ^2 f(x_k) \preceq G_k \preceq \xi _k \frac{L}{\mu } \nabla ^2 f(x_k), \end{aligned}$$

33 $$\begin{aligned}&\frac{1}{\xi _{k+1}} J_k \preceq G_k \preceq \xi _{k+1} \frac{L}{\mu } J_k. \end{aligned}$$

### Proof

For $k=0$, (32) follows from (26) and the fact that $G_0 = L B$ and $\xi _0 = 1$. Now suppose that $k \ge 0$, and that (32) has already been proved for all indices up to *k*. Then, applying Lemma 5.1 to (32), we obtain

34 $$\begin{aligned} \frac{1}{\xi _k \left( 1 + \frac{M r_k}{2} \right) } J_k \preceq G_k \preceq \left( 1 + \frac{M r_k}{2} \right) \xi _k \frac{L}{\mu } J_k. \end{aligned}$$

Since $(1 + \frac{M r_k}{2}) \xi _k \le \xi _{k+1}$ by (31), this proves (33) for the index *k*. Applying Lemma 3.1 to (34), we get $\frac{1}{\xi _k( 1 + \frac{M r_k}{2})} J_k \preceq G_{k+1} \preceq (1 + \frac{M r_k}{2} ) \xi _k \frac{L}{\mu } J_k$, and so

$$\begin{aligned} \begin{array}{rcl} G_{k+1} &{}{\mathop {\preceq }\limits ^{(29)}}&{} \left( 1 + \frac{M r_k}{2} \right) ^2 \xi _k \frac{L}{\mu } \nabla ^2 f(x_{k+1}) \;{\mathop {\preceq }\limits ^{(31)}}\; \xi _{k+1} \frac{L}{\mu } \nabla ^2 f(x_{k+1}), \\ G_{k+1} &{}{\mathop {\succeq }\limits ^{(29)}}&{} \frac{1}{\left( 1 + \frac{M r_k}{2} \right) ^2 \xi _k} \nabla ^2 f(x_{k+1}) \;{\mathop {\succeq }\limits ^{(31)}}\; \frac{1}{\xi _{k+1}} \nabla ^2 f(x_{k+1}). \end{array} \end{aligned}$$

This proves (32) for the index $k+1$, and we can continue by induction.$\square$

### Corollary 5.1

For all $k \ge 0$, we have

35 $$\begin{aligned} r_k \le \xi _k \lambda _k. \end{aligned}$$

### Proof

Indeed,

$$\begin{aligned} \qquad \qquad \begin{array}[b]{rcl} r_k \;{\mathop {=}\limits ^{(31)}}\; \Vert u_k \Vert _{x_k} &{}{\mathop {=}\limits ^{(35)}}&{} \langle \nabla f(x_k), G_k^{-1} \nabla ^2 f(x_k) G_k^{-1} \nabla f(x_k) \rangle ^{1/2} \\ &{}{\mathop {\le }\limits ^{(32)}}&{} \xi _k \langle \nabla f(x_k), \nabla ^2 f (x_k)^{-1} \nabla f(x_k) \rangle ^{1/2} \;{\mathop {=}\limits ^{(30)}}\; \xi _k \lambda _k.\qquad \qquad \square \end{array} \end{aligned}$$

The second step in our analysis is to establish a preliminary version of the linear convergence theorem for the scheme ().

### Lemma 5.3

For all $k \ge 0$, we have

36 $$\begin{aligned} \lambda _k \le \sqrt{\xi _k} \lambda _0 \prod \limits _{i=0}^ {k-1} q_i, \end{aligned}$$

where

37 $$\begin{aligned} q_i :=\max \left\{ 1 - \frac{\mu }{\xi _{i+1} L}, \xi _ {i+1} - 1 \right\} . \end{aligned}$$

### Proof

Let $k, i \ge 0$ be arbitrary. By Taylor’s formula, we have

38 $$\begin{aligned} \nabla f(x_{i+1}) {\mathop {=}\limits ^{(25)}} \nabla f(x_i) + J_i u_i \;{\mathop {=}\limits ^{(25)}}\; J_i (J_i^{-1} - G_i^{-1}) \nabla f(x_i). \end{aligned}$$

Hence,

39 $$\begin{aligned} \begin{array}{rcl} \lambda _{i+1} &{}{\mathop {=}\limits ^{(30)}}&{} \langle \nabla f(x_{i+1}), \nabla ^2 f(x_{i+1})^{-1} \nabla f(x_{i+1}) \rangle ^{1/2} \\ &{}{\mathop {\le }\limits ^{(29)}}&{} \sqrt{1 + \frac{M r_i}{2}} \langle \nabla f (x_{i+1}), J_i^{-1} \nabla f(x_{i+1}) \rangle ^{1/2} \\ &{}{\mathop {=}\limits ^{(38)}}&{} \sqrt{1 + \frac{M r_i}{2}} \langle \nabla f (x_i), (J_i^{-1} - G_i^{-1}) J_i (J_i^{-1} - G_i^{-1}) \nabla f(x_i) \rangle ^{1/2}. \end{array} \end{aligned}$$

Note that $-(\xi _{i+1} - 1) J_i^{-1} {\mathop {\preceq }\limits ^{(33)}} J_i^{-1} - G_i^{-1} {\mathop {\preceq }\limits ^{(33)}} \left( 1 - \frac{\mu }{\xi _{i+1} L} \right) J_i^{-1}$. Therefore,

$$\begin{aligned} (J_i^{-1} - G_i^{-1}) J_i (J_i^{-1} - G_i^{-1}) {\mathop {\preceq }\limits ^{(37)}} q_i^2 J_i^{-1} \;{\mathop {\preceq }\limits ^{(28)}}\; q_i^2 \left( 1 + \frac{M r_i}{2} \right) \nabla ^2 f(x_i)^{-1}. \end{aligned}$$

Thus, $\lambda _{i+1} \le \left( 1 + \frac{M r_i}{2}\right) q_i \lambda _i$ in view of (39) and (30). Consequently,

$$\begin{aligned} \lambda _k \le \lambda _0 \prod \limits _{i=0}^{k-1} \left( 1 + \frac{M r_i}{2} \right) q_i \;\le \; \lambda _0 \prod \limits _{i=0}^{k-1} e^{\frac{M r_i}{2}} q_i \;{\mathop {=}\limits ^{(31)}}\; \sqrt{\xi _k} \lambda _0 \prod \limits _{i=0}^{k-1} q_i.\qquad \qquad \ \square \end{aligned}$$

Next, we establish a preliminary version of the theorem on superlinear convergence of the scheme (). The proof uses the augmented log-det barrier potential function and is essentially a generalization of the corresponding proof of Theorem 4.3.

### Lemma 5.4

For all $k \ge 1$, we have

40 $$\begin{aligned} \lambda _k \le \left[ \frac{1 + \xi _k}{\prod _{i=0}^{k-1} (\tau _i \frac{\mu }{\xi _{i+1}^2 L} + 1 - \tau _i)^{1/k}} \left( e^{\frac{13}{6} \frac{n}{k} \ln \left( \xi _{k+1}^{\xi _{k+1}} \frac{L}{\mu } \right) } - 1 \right) \right] ^{k/2} \sqrt{\xi _k \frac{L}{\mu }} \cdot \lambda _0. \end{aligned}$$

### Proof

Without loss of generality, assume that $u_i \ne 0$ for all $0 \le i \le k$. Denote $\psi _i :=\psi (G_i, J_i)$, $\tilde{\psi }_{i+1} :=\psi (G_{i+1}, J_i)$, $\nu _i :=\nu (J_i, G_i, u_i)$, $p_i :=\tau _i \frac{\mu }{\xi _{i+1}^2 L} + 1 - \tau _i$, and $g_i :=\Vert \nabla f(x_i) \Vert _{G_i}^*$ for any $0 \le i \le k$.

Let $0 \le i \le k - 1$ be arbitrary. By Lemma 3.4 and (33), we have

41 $$\begin{aligned} \frac{6}{13} \ln \left( 1 + p_i \nu _i^2 \right) \le \psi _i - \tilde{\psi }_{i+1} \;=\; \psi _i - \psi _{i+1} + \varDelta _i, \end{aligned}$$

where

42 $$\begin{aligned} \varDelta _i :=\psi _{i+1} - \tilde{\psi }_{i+1} \;{\mathop {=}\limits ^{(8)}}\; \langle G_{i+1}^{-1}, J_{i+1} - J_i \rangle + \ln \mathrm{Det}(J_{i+1}^{-1}, J_i). \end{aligned}$$

Note that $J_i \succeq (1 + \frac{M r_i}{2})^{-1} \nabla ^2 f(x_{i+1}) \succeq (1 + \frac{M r_i}{2})^ {-1} (1 + \frac{M r_{i+1}}{2})^{-1} J_{i+1}$ in view of (29) and (28). In particular, $J_i \succeq e^{-\frac{M}{2} (r_i + r_{i+1})} J_{i+1} \succeq (1 - \frac{M}{2} (r_i + r_{i+1})) J_{i+1}$. Therefore, $J_{i+1} - J_i \preceq \frac{M}{2} (r_i + r_ {i+1}) J_{i+1}$, and so

$$\begin{aligned} \begin{array}{rcl} \sum \limits _{i=0}^{k-1} \langle G_{i+1}^{-1}, J_{i+1} - J_i \rangle &{}\le &{} \frac{M}{2} \sum \limits _{i=0}^{k-1} (r_i + r_{i+1}) \langle G_{i+1}^{-1}, J_{i+1} \rangle \\ &{}{\mathop {\le }\limits ^{(33)}}&{} n \frac{M}{2} \sum \limits _{i=0}^{k-1} \xi _{i+2} (r_i + r_{i+1}) \;{\mathop {\le }\limits ^{(31)}}\; n \xi _{k+1} \frac{M}{2} \sum \limits _{i=0}^{k-1} (r_i + r_{i+1}) \\ &{}\le &{} n \xi _{k+1} M \sum \limits _{i=0}^k r_i \;{\mathop {=}\limits ^{(31)}}\; n \xi _{k+1} \ln \xi _{k+1}. \end{array} \end{aligned}$$

Consequently,

43 $$\begin{aligned} \sum \limits _{i=0}^{k-1} \varDelta _i {\mathop {\le }\limits ^{(42)}} n \xi _ {k+1} \ln \xi _{k+1} + \ln \mathrm{Det}(J_k^{-1}, J_0). \end{aligned}$$

Summing up (41), we thus obtain

$$\begin{aligned} \begin{array}{rcl} &{}&{}\frac{6}{13} \sum \limits _{i=0}^{k-1} \ln (1 + p_i \nu _i^2) \le \psi _0 - \psi _k + \sum \limits _{i=0}^{k-1} \varDelta _i \;{\mathop {\le }\limits ^{(9)}}\; \psi _0 + \sum \limits _{i=0}^{k-1} \varDelta _i \\ &{}{\mathop {=}\limits ^{(8)}}&{} \ln \mathrm{Det}(J_0^{-1}, L B) - \langle \frac{1}{L} B^{-1}, L B - J_0 \rangle + \sum \limits _{i=0}^{k-1} \varDelta _i \\ &{}{\mathop {\le }\limits ^{(43)}}&{} \ln \mathrm{Det}(J_k^{-1}, L B) - \langle \frac{1}{L} B^{-1}, L B - J_0 \rangle + n \xi _{k+1} \ln \xi _{k+1} \\ &{}{\mathop {\le }\limits ^{(26)}}&{} n \ln \frac{L}{\mu } + n \xi _{k+1} \ln \xi _{k+1} \;=\; n \ln \left( \xi _{k+1}^{\xi _{k+1}} \frac{L}{\mu } \right) . \end{array} \end{aligned}$$

By the convexity of function $t \mapsto \ln (1 + e^t)$, it follows that

44 $$\begin{aligned} \begin{array}{rcl} &{}&{}\frac{13}{6} \frac{n}{k} \ln \left( \xi _ {k+1}^{\xi _{k+1}} \frac{L}{\mu } \right) \;\ge \; \frac{1}{k} \sum \limits _{i=0}^{k-1} \ln (1 + p_i \nu _i^2) \;=\; \frac{1}{k} \sum \limits _{i=0}^{k-1} \ln (1 + e^{\ln (p_i \nu _i^2)}) \\ &{}\ge &{} \ln \left( 1 + e^{\frac{1}{k} \sum _{i=0}^ {k-1} \ln (p_i \nu _i^2)} \right) \;=\; \ln \left( 1 + \left[ \prod \limits _{i=0}^{k-1} p_i \nu _i^2 \right] ^{1/k} \right) . \end{array} \end{aligned}$$

At the same time, $\nu _i^2 \ge \frac{1}{1 + \xi _{i+1}} \frac{\langle (G_i - J_i) G_{i+1}^{-1} (G_i - J_i) u_i, u_i \rangle }{\langle G_i u_i, u_i \rangle } = \frac{1}{1 + \xi _ {i+1}} \frac{g_{i+1}^2}{g_i^2}$ in view of Lemma 3.5, (33) and since $G_i u_i = -\nabla f(x_i)$, $J_i u_i = \nabla f(x_ {i+1}) - \nabla f(x_i)$. Hence, we can write $\prod _{i=0}^{k-1} \nu _i^2 \ge \frac{g_k^2}{g_0^2} \prod _{i=0}^{k-1} \frac{1}{1 + \xi _{i+1}} {\mathop {\ge }\limits ^{(31)}} \frac{1}{(1 + \xi _k)^k} \frac{g_k^2}{g_0^2}$. Consequently, $\frac{13}{6} \frac{n}{k} \ln ( \xi _{k+1}^{\xi _{k+1}} \frac{L}{\mu }) {\mathop {\ge }\limits ^{(44)}} \ln \left( 1 + \frac{\prod _{i=0}^{k-1} p_i^ {1/k}}{1 + \xi _k} \left[ \frac{g_k}{g_0} \right] ^{2/k} \right)$. Rearranging, we obtain that $g_k \le \left[ \frac{1 + \xi _k}{\prod _{i=0}^{k-1} p_i^{1/k}} (e^{\frac{13}{6} \frac{n}{k} \ln ( \xi _ {k+1}^{\xi _{k+1}} \frac{L}{\mu } )} - 1 ) \right] ^{k/2} g_0$. But $\lambda _k \le \sqrt{\xi _k \frac{L}{\mu }} \cdot g_k$ by (32), and $g_0 \le \lambda _0$ in view of (26) and the fact that $G_0 = L B$.$\square$

In the quadratic case ($M = 0$), we have $\xi _k \equiv 1$ (see (31)), and Lemmas 5.2 and 5.3 reduce to the already known Theorem 4.1, and Lemma 5.4 reduces to the already known Theorem 4.2. In the general case, the quantities $\xi _k$ can grow with iterations. However, as we will see in a moment, by requiring the initial point $x_0$ in the scheme () to be sufficiently close to the solution, we can still ensure that $\xi _k$ stay *uniformly bounded* by a sufficiently small absolute constant. This allows us to recover all the main results of the quadratic case.

To write down the region of local convergence of (), we need to introduce one more quantity, related to the starting moment of superlinear convergence^3^:

45 $$\begin{aligned} K_0 :=\left\lceil \frac{1}{\tau \frac{4 \mu }{9 L} + 1 - \tau } 8 n \ln \frac{2 L}{\mu } \right\rceil , \qquad \tau :=\sup \limits _{k \ge 0} \tau _k \quad (\;\le \; 1). \end{aligned}$$

For DFP ($\tau _k \equiv 1$) and BFGS ($\tau _k \equiv 0)$, we have, respectively,

46 $$\begin{aligned} K_0^{\mathrm{DFP}} = \left\lceil \frac{18 n L}{\mu } \ln \frac{2 L}{\mu } \right\rceil , \qquad K_0^{\mathrm{BFGS}} = \left\lceil 8 n \ln \frac{2 L}{\mu } \right\rceil . \end{aligned}$$

Now we are ready to prove the main result of this section.

### Theorem 5.1

Suppose that, in scheme (), we have

47 $$\begin{aligned} M \lambda _0 \le \frac{\ln \frac{3}{2}}{\left( \frac{3}{2} \right) ^{\frac{3}{2}}} \max \left\{ \frac{\mu }{2 L}, \frac{1}{K_0 + 9} \right\} . \end{aligned}$$

Then, for all $k \ge 0$,

48 $$\begin{aligned}&\frac{2}{3} \nabla ^2 f(x_k) \preceq G_k \preceq \frac{3 L}{2 \mu } \nabla ^2 f(x_k), \end{aligned}$$

49 $$\begin{aligned}&\lambda _k \le \left( 1 - \frac{\mu }{2 L} \right) ^k \sqrt{\frac{3}{2}} \cdot \lambda _0, \end{aligned}$$

and, for all $k \ge 1$,

50 $$\begin{aligned} \lambda _k \le \left[ \frac{5}{2 \prod _{i=0}^{k-1} (\tau _i \frac{4 \mu }{9 L} + 1 - \tau _i)^{1/k}} \left( e^{\frac{13}{6} \frac{n}{k} \ln \frac{2 L}{\mu } } - 1 \right) \right] ^{k/2} \sqrt{\frac{3 L}{2 \mu }} \cdot \lambda _0. \end{aligned}$$

### Proof

Let us prove by induction that, for all $k \ge 0$, we have

51 $$\begin{aligned} \xi _k \le \frac{3}{2}. \end{aligned}$$

Clearly, (51) is satisfied for $k=0$ since $\xi _0 = 1$. It is also satisfied for $k=1$ since $\xi _1 {\mathop {=}\limits ^{(31)}} e^{M r_0} {\mathop {\le }\limits ^{(35)}} e^{\xi _0 M \lambda _0} {\mathop {=}\limits ^{(31)}} e^{M \lambda _0} {\mathop {\le }\limits ^{(47)}} \frac{3}{2}$.

Now let $k \ge 0$, and suppose that (51) has already been proved for all indices up to $k+1$. Then, applying Lemma 5.2, we obtain (48) for all indices up to $k+1$. Applying now Lemma 5.3 and using for all $0 \le i \le k$ the relation $q_i {\mathop {=}\limits ^{(37)}} \max \{ 1 - \frac{\mu }{\xi _{i+1} L}, \xi _{i+1} - 1 \} {\mathop {\le }\limits ^{(51)}} \max \{1 - \frac{2 \mu }{3 L}, \frac{1}{2} \} \le 1 - \frac{\mu }{2 L}$, we obtain (49) for all indices up to $k+1$. Finally, if $k \ge 1$, then, applying Lemma 5.4 and using that $\xi _{i+1}^{\xi _{i+1}} {\mathop {\le }\limits ^{(51)}} ( \frac{3}{2} )^{\frac{3}{2}} = \frac{3}{2} \sqrt{\frac{3}{2}} \le \frac{3}{2} (1 + \frac{1}{4}) = \frac{15}{8} \le 2$ for all $0 \le i \le k$, we obtain (50) for all indices up to *k*. Thus, at this moment, (48) and (49) are proved for all indices up to $k+1$, while (50) is proved only up to *k*.

To finish the inductive step, it remains to prove that (51) is satisfied for the index $k+2$, or, equivalently, in view of (31), that $M \sum _{i=0}^{k+1} r_i \le \ln \frac{3}{2}$. Since $M \sum _{i=0}^{k+1} r_i \le M \sum _{i=0}^{k+1} \xi _i \lambda _i \le \frac{3}{2} M \sum _{i=0}^{k+1} \lambda _i$ in view of (35) and (51), respectively, it suffices to show that $\frac{3}{2} M \sum _{i=0}^{k+1} \lambda _i \le \ln \frac{3}{2}$.

Note that

52 $$\begin{aligned} \frac{3}{2} M \sum \limits _{i=0}^{k+1} \lambda _i {\mathop {\le }\limits ^{(49)}} \left( \frac{3}{2} \right) ^{\frac{3}{2}} M \lambda _0 \sum \limits _{i=0}^{k+1} \left( 1 - \frac{\mu }{2 L} \right) ^i \;\le \; \left( \frac{3}{2} \right) ^{\frac{3}{2}} \frac{2 L}{\mu } M \lambda _0. \end{aligned}$$

Therefore, if we could prove that

53 $$\begin{aligned} \frac{3}{2} M \sum \limits _{i=0}^{k+1} \lambda _i \le \left( \frac{3}{2} \right) ^{\frac{3}{2}} (K_0 + 9) M \lambda _0,\end{aligned}$$

then, combining (52) and (53), we would obtain

$$\begin{aligned} \frac{3}{2} M \sum \limits _{i=0}^{k+1} \lambda _i \le \left( \frac{3}{2} \right) ^{\frac{3}{2}} \min \left\{ \frac{2 L}{\mu }, K_0 + 9 \right\} M \lambda _0 \;{\mathop {\le }\limits ^{(47)}}\; \ln \frac{3}{2}, \end{aligned}$$

which is exactly what we need. Let us prove (53). If $k \le K_0$, in view of (49), we have $\frac{3}{2} M \sum _{i=0}^{k+1} \lambda _i \le \left( \frac{3}{2} \right) ^{\frac{3}{2}} (k + 2) M \lambda _0 \le \left( \frac{3}{2} \right) ^{\frac{3}{2}} (K_0 + 2) M \lambda _0$, and (53) follows. Therefore, from now on, we can assume that $k \ge K_0$. Then^4^,

$$\begin{aligned} \begin{array}{rcl} \frac{3}{2} M \sum \limits _{i=0}^{k+1} \lambda _i &{}=&{} \frac{3}{2} M \left( \sum \limits _{i=0}^{K_0-1} \lambda _i + \lambda _ {k+1} \right) + \frac{3}{2} M \sum \limits _{i=K_0}^k \lambda _i \\ &{}{\mathop {\le }\limits ^{(49)}}&{} \left( \frac{3}{2} \right) ^{\frac{3}{2}} (K_0 + 1) M \lambda _0 + \frac{3}{2} M \sum \limits _{i=K_0}^k \lambda _i. \end{array} \end{aligned}$$

It remains to show $\frac{3}{2} M \sum _{i=K_0}^k \lambda _i \le \left( \frac{3}{2} \right) ^{\frac{3}{2}} 8 M \lambda _0$. We can do this using (50).

First, let us make some estimations. Clearly, for all $0< t < 1$, we have $e^t = \sum _{j=0}^\infty \frac{t^j}{j!} \le 1 + t + \frac{t^2}{2} \sum _{j=0}^\infty t^j = 1 + t ( 1 + \frac{t}{2 (1 - t)})$. Hence, for all $0 < t \le 1$, we obtain $e^{\frac{13 t}{48}} - 1 \le \frac{13 t}{48} ( 1 + \frac{\frac{13}{48}}{2 (1 - \frac{13}{48})} ) = \frac{13 t}{48} \cdot \frac{83}{70} \le \frac{13 t}{48} \cdot \frac{6}{5} = \frac{13 t}{40}$, and so

54 $$\begin{aligned} \left[ \frac{5}{2 t} \left( e^{\frac{13 t}{48}} - 1 \right) \right] ^{1/2} \le \sqrt{ \frac{5}{2 t} \cdot \frac{13 t}{40}} \;=\; \sqrt{\frac{13}{16}} \;\le \; \frac{11}{12}. \end{aligned}$$

At the same time, $\frac{11}{12} = 1 - \frac{1}{12} \le e^{-\frac{1}{12}}$. Hence,

55 $$\begin{aligned} \begin{array}{rcl} \left( \frac{11}{12} \right) ^{K_0} \sqrt{\frac{L}{\mu }} &{}{\mathop {\le }\limits ^{(45)}}&{} \left( \frac{11}{12} \right) ^{8 \ln \frac{2 L}{\mu }} \sqrt{\frac{L}{\mu }} \;\le \; e^ {-\frac{2}{3} \ln \frac{2 L}{\mu }} \sqrt{\frac{L}{\mu }} \;=\; \left( \frac{2 L}{\mu } \right) ^{-\frac{2}{3}} \sqrt{ \frac{L}{\mu }} \\ &{}=&{} 2^{-\frac{2}{3}} \left( \frac{L}{\mu } \right) ^{- \frac{1}{6}} \;\le \; 2^{-\frac{2}{3}} \;\le \; \frac{2}{3}. \end{array} \end{aligned}$$

Thus, for all $K_0 \le i \le k$, and $p :=\tau \frac{4 \mu }{9 L} + 1 - \tau \;{\mathop {\le }\limits ^{(45)}}\; \prod _ {j=0}^{i-1} (\tau _i \frac{4 \mu }{9 L} + 1 - \tau _i)^{1/i}$:

$$\begin{aligned} \begin{array}{rcl} \lambda _i &{}{\mathop {\le }\limits ^{(50)}}&{} \left[ \frac{5}{2 p} \left( e^{ \frac{13}{6} \frac{n}{i} \ln \frac{2 L}{\mu } } - 1 \right) \right] ^{i/2} \sqrt{\frac{3 L}{2 \mu }} \cdot \lambda _0 \\ &{}{\mathop {\le }\limits ^{(45)}}&{} \left[ \frac{5}{2 p} \left( e^{\frac{13 p}{48}} - 1 \right) \right] ^{i/2} \sqrt{\frac{3 L}{2 \mu }} \cdot \lambda _0 \;{\mathop {\le }\limits ^{(54)}}\; \left( \frac{11}{12} \right) ^i \sqrt{\frac{3 L}{2 \mu }} \cdot \lambda _0 \\ &{}=&{} \left( \frac{11}{12} \right) ^{i-K_0} \left( \frac{11}{12} \right) ^{K_0} \sqrt{\frac{3 L}{2 \mu }} \cdot \lambda _0 \;{\mathop {\le }\limits ^{(55)}}\; \left( \frac{11}{12} \right) ^{i-K_0} \frac{2}{3} \cdot \sqrt{\frac{3}{2}} \cdot \lambda _0. \end{array} \end{aligned}$$

Hence, $$\frac{3}{2} M \sum _{i=K_0}^k \lambda _i \le (\frac{3}{2})^{\frac{3}{2}} M \lambda _0 \cdot \frac{2}{3} \sum _{i=K_0}^k (\frac{11}{12})^ {i-K_0} \le (\frac{3}{2})^{ \frac{3}{2}} 8 M \lambda _0$$.$$\square $$

### Remark 5.2

In accordance with Theorem 5.1, the parameter *M* of strong self-concordancy affects only the size of the region of local convergence of the process (), and not its rate of convergence. We do not know whether this is an artifact of the analysis or not, but it might be an interesting topic for future research. For a quadratic function, we have $M = 0$, and so the scheme () is globally convergent.

The region of local convergence, specified by (47), depends on the *maximum* of two quantities: $\frac{\mu }{L}$ and $\frac{1}{K_0}$. For DFP, the $\frac{1}{K_0}$ part in this maximum is in fact redundant, and its region of local convergence is simply inversely proportional to the condition number: $O\left( \frac{\mu }{L} \right)$. However, for BFGS, the $\frac{1}{K_0}$ part does not disappear, and we obtain the following region of local convergence:

$$\begin{aligned} M \lambda _0 \le \max \left\{ O\left( \frac{\mu }{L}\right) , \ O\left( \frac{1}{n \ln \frac{2 L}{\mu }} \right) \right\} . \end{aligned}$$

Clearly, the latter region can be much bigger than the former when the condition number $\frac{L}{\mu }$ is significantly larger than the dimension *n*.

### Remark 5.3

The previous estimate of the size of the region of local convergence, established in [27], was $O(\frac{\mu }{L})$ for both DFP and BFGS.

### Example 5.1

Consider the functions

$$\begin{aligned} f(x) :=f_0(x) + \frac{\mu }{2} \Vert x \Vert ^2, \qquad f_0(x) :=\ln \left( \sum \limits _{i=1}^m e^{\langle a_i, x \rangle + b_i} \right) , \qquad x \in \mathbb {E}, \end{aligned}$$

where $a_i \in \mathbb {E}^*$, $b_i \in \mathbb {R}$, $i = 1, \ldots , m$, $\mu > 0$, and $\Vert \cdot \Vert$ is the Euclidean norm, induced by the operator *B*. Let $\gamma > 0$ be such that

$$\begin{aligned} \Vert a_i \Vert _* \le \gamma , \qquad i = 1, \ldots , m, \end{aligned}$$

where $\Vert \cdot \Vert _*$ is the norm conjugate to $\Vert \cdot \Vert$. Define

$$\begin{aligned} \pi _i(x) :=\frac{e^{\langle a_i, x \rangle + b_i}}{\sum _{j=1}^m e^{\langle a_j, x \rangle + b_j}}, \qquad x \in \mathbb {E}, \quad i = 1, \ldots , m. \end{aligned}$$

Clearly, $\sum _{i=1}^m \pi _i(x) = 1$, $\pi _i(x) > 0$ for all $x \in \mathbb {E}$, $i = 1, \ldots , m$. It is not difficult to check that, for all $x, h \in \mathbb {E}$, we have^5^

$$\begin{aligned} \begin{array}{rcl} \langle \nabla f_0(x), h \rangle &{}=&{} \sum \limits _{i=1}^m \pi _i(x) \langle a_i, h \rangle \;\le \; \gamma .\\ \langle \nabla ^2 f_0(x) h, h \rangle &{}=&{} \sum \limits _{i=1}^m \pi _i(x) \langle a_i - \nabla f_0(x), h \rangle ^2 \\ &{}=&{} \sum \limits _{i=1}^m \pi _i(x) \langle a_i, h \rangle ^2 - \langle \nabla f_0(x), h \rangle ^2 \;\le \; \gamma ^2 \Vert h \Vert ^2, \\ D^3 f_0(x) [h, h, h] &{}=&{} \sum \limits _{i=1}^m \pi _i(x) \langle a_i - \nabla f_0(x), h \rangle ^3 \\ &{}\le &{} 2 \gamma \Vert h \Vert \langle \nabla ^2 f_0(x) h, h \rangle \;\le \; 2 \gamma ^3 \Vert h \Vert ^3. \end{array} \end{aligned}$$

Thus, $f_0$ is a convex function with $\gamma ^2$-Lipschitz gradient and $(2 \gamma ^3)$-Lipschitz Hessian. Consequently, the function *f* is $\mu$-strongly convex with *L*-Lipschitz gradient, $(2 \gamma ^3)$-Lipschitz Hessian, and, in view of [26, Example 4.1], *M*-strongly self-concordant, where

$$\begin{aligned} L :=\gamma ^2 + \mu , \qquad M:= & {} \frac{2 \gamma ^3}{\mu ^{3/2}}. \end{aligned}$$

Let the regularization parameter $\mu$ be sufficiently small, namely $\mu \le \gamma ^2$. Denote $Q :=\frac{\gamma ^2}{\mu } \ge 1$. Then, $Q \le \frac{L}{\mu } \le 2 Q$, $M = 2 Q^{3/2}$, so, according to (47), the region of local convergence of BFGS can be described as follows:

$$\begin{aligned} \lambda _0 \le \max \left\{ O\left( \frac{1}{Q^{5/2}} \right) , O\left( \frac{1}{n Q^{3/2} \ln (4 Q)} \right) \right\} .\qquad \qquad \qquad \qquad \ \ \square \end{aligned}$$

## Discussion

Let us compare the new convergence rates, obtained in this paper for the classical DFP and BFGS methods, with the previously known ones from [27]. Since the estimates for the general nonlinear case differ from those for the quadratic one just in absolute constants, we only discuss the latter case.

In what follows, we use our standard notation: *n* is the dimension of the space, $\mu$ is the strong convexity parameter, *L* is the Lipschitz constant of the gradient, and $\lambda _k$ is the local norm of the gradient at the *k*th iteration.

For BFGS, the previously known rate (see [27, Theorem 3.2]) is

56 $$\begin{aligned} \lambda _k \le \left( \frac{n L}{\mu k} \right) ^{k/2} \lambda _0. \end{aligned}$$

Although (56) is formally valid for all $k \ge 1$, it becomes useful^6^ only after

57 $$\begin{aligned} \widehat{K}_0^{\mathrm{BFGS}} :=\frac{n L}{\mu } \end{aligned}$$

iterations. Thus, $\widehat{K}_0^{\mathrm{BFGS}}$ can be thought of as the *starting moment* of the superlinear convergence, according to the estimate (56).

In this paper, we have obtained a new estimate (Theorem 4.2):

58 $$\begin{aligned} \lambda _k \le \left[ 2 \left( e^{\frac{n}{k} \ln \frac{L}{\mu }} - 1 \right) \right] ^{k/2} \sqrt{\frac{L}{\mu }} \cdot \lambda _0. \end{aligned}$$

Its starting moment of superlinear convergence can be described as follows:

59 $$\begin{aligned} K_0^{\mathrm{BFGS}} :=4 n \ln \frac{L}{\mu }. \end{aligned}$$

Indeed, since $e^t \le \frac{1}{1-t} = 1 + \frac{t}{1-t}$ for any $t < 1$, we have, for all $k \ge K_0^{\mathrm{BFGS}}$,

60 $$\begin{aligned} e^{\frac{n}{k} \ln \frac{L}{\mu }} - 1 \le \frac{\frac{n}{k} \ln \frac{L}{\mu }}{1 - \frac{n}{k} \ln \frac{L}{\mu }} \;{\mathop {\le }\limits ^{(59)}}\; \frac{\frac{n}{k} \ln \frac{L}{\mu }}{1 - \frac{1}{4}} \;=\; \frac{4 n}{3 k} \ln \frac{L}{\mu }. \end{aligned}$$

At the same time, for all $k \ge K_0^{\mathrm{BFGS}}$:

61 $$\begin{aligned} \sqrt{\frac{L}{\mu }} = e^{\frac{1}{2} \ln \frac{L}{\mu }} \;{\mathop {\le }\limits ^{(59)}}\; e^{\frac{k}{8}} \;=\; (e^{\frac{1}{4}} )^ {k/2} \;\le \; \left( \frac{4}{3} \right) ^{k/2} \;\le \; \left( \frac{3}{2} \right) ^{k/2}. \end{aligned}$$

Hence, according the new estimate (58), for all $k \ge K_0^{\mathrm{BFGS}}$:

62 $$\begin{aligned} \lambda _k {\mathop {\le }\limits ^{(60)}} \left( \frac{8 n}{3 k} \ln \frac{L}{\mu } \right) ^{k/2} \sqrt{\frac{L}{\mu }} \cdot \lambda _0 \;{\mathop {\le }\limits ^{(61)}}\; \left( \frac{4 n}{k} \ln \frac{L}{\mu } \right) ^{k/2} \lambda _0 \qquad (\;{\mathop {\le }\limits ^{(59)}}\; \lambda _0). \end{aligned}$$

Comparing the previously known efficiency estimate (56) and its starting moment of superlinear convergence (57) with the new ones (62), (59), we thus conclude that we manage to put the condition number $\frac{L}{\mu }$ *under the logarithm*.

For DFP, the previously known rate (see [27, Theorem 3.2]) is

$$\begin{aligned} \lambda _k \le \left( \frac{n L^2}{\mu ^2 k} \right) ^{k/2} \lambda _0 \end{aligned}$$

with the following starting moment of the superlinear convergence:

63 $$\begin{aligned} \widehat{K}_0^{\mathrm{DFP}} :=\frac{n L^2}{\mu ^2}. \end{aligned}$$

The new rate, which we have obtained in this paper (Theorem 4.2), is

64 $$\begin{aligned} \lambda _k \le \left[ \frac{2 L}{\mu } \left( e^{\frac{n}{k} \ln \frac{L}{\mu }} - 1 \right) \right] ^{k/2} \sqrt{ \frac{L}{\mu }} \cdot \lambda _0. \end{aligned}$$

Repeating the same reasoning as above, we can easily obtain that the new starting moment of the superlinear convergence can be described as follows:

65 $$\begin{aligned} K_0^{\mathrm{DFP}} :=\frac{4 n L}{\mu } \ln \frac{L}{\mu }, \end{aligned}$$

and, for all $k \ge K_0^{\mathrm{DFP}}$, the new estimate (64) takes the following form:

$$\begin{aligned} \lambda _k \le \left( \frac{4 n L}{\mu k} \ln \frac{L}{\mu } \right) ^{k/2} \lambda _0 \quad (\;{\mathop {\le }\limits ^{(65)}}\; \lambda _0). \end{aligned}$$

Thus, compared to the old result, we have improved the factor $\frac{L^2}{\mu ^2}$ up to $\frac{L}{\mu } \ln \frac{L}{\mu }$. Interestingly enough, the ratio between the old starting moments (63), (57) of the superlinear convergence of DFP and BFGS and the new ones (65), (59) have remained the same, $\frac{L}{\mu }$, although the both estimates have been improved.

It is also interesting whether the results, obtained in this paper, can be applied to *limited-memory* quasi-Newton methods such as L-BFGS [30]. Unfortunately, it seems like the answer is negative. The main problem is that we cannot say anything interesting about just a *few* iterations of BFGS. Indeed, according to our main result, after *k* iterations of BFGS, the initial residual is contracted by the factor of the form $[ \exp (\frac{n}{k} \ln \frac{L}{\mu }) - 1]^k$. For all values $k \le n \ln \frac{L}{\mu }$, this contraction factor is in fact bigger than 1, so the result becomes useless.

## Conclusions

We have presented a new theoretical analysis of local superlinear convergence of classical quasi-Newton methods from the convex Broyden class. Our analysis has been based on the potential function involving the logarithm of determinant of Hessian approximation and the trace of inverse Hessian approximation. Compared to the previous works, we have obtained new convergence rate estimates, which have much better dependency on the condition number of the problem.

Note that all our results are *local*, i.e. they are valid under the assumption that the starting point is sufficiently close to a minimizer. In particular, there is no contradiction between our results and the fact that the DFP method is not known to be globally convergent with inexact line search (see, e.g., [31]).

Let us mention several open questions. First, looking at the starting moment of superlinear convergence of the BFGS method, in addition to the dimension of the problem, we see the presence of the logarithm of its condition number. Although typically such logarithmic factors are considered small, it is still interesting to understand whether this factor can be completely removed.

Second, all the superlinear convergence rates, which we have obtained for the convex Broyden class in this paper, are expressed in terms of the parameter $\tau$, which controls the weight of the DFP component in the updating formula for the *inverse* operator. At the same time, in [27], the corresponding estimates were presented in terms of the parameter $\phi$, which controls the weight of the DFP component in the updating formula for the *primal* operator. Of course, for the extreme members of the convex Broyden class, DFP and BFGS, $\phi$ and $\tau$ coincide. However, in general, they could be quite different. We do not know if it is possible to express the results of this paper in terms of $\phi$ instead of $\tau$.

Finally, in all the methods, which we considered, the initial Hessian approximation $G_0$ was *LB*, where *L* is the Lipschitz constant of the gradient, measured relative to the operator *B*. We always assume that this constant is known. Of course, it is interesting to develop some *adaptive* algorithms, which could start from any initial guess $L_0$ for the constant *L*, and then somehow dynamically adjust the Hessian approximations in iterations, yet retaining all the original efficiency estimates.

## Appendix

### Lemma A.1

Let $A, G : \mathbb {E}\rightarrow \mathbb {E}^*$ be self-adjoint positive definite linear operators, let $u \in \mathbb {E}$ be nonzero, and let $\tau \in \mathbb {R}$ be such that $G_+ :=\mathrm{Broyd}_{\tau }(A, G, u)$ is well defined. Then,

66 $$\begin{aligned} \begin{array}{rcl} G_+^{-1} &{}=&{} \tau \left[ G^{-1} - \frac{G^{-1} A u u^* A G^{-1}}{\langle A G^{-1} A u, u \rangle } + \frac{u u^*}{\langle A u, u \rangle } \right] \\ &{}&{}\quad + \ (1 - \tau ) \left[ G^{-1} - \frac{G^{-1} A u u^* + u u^* A G^{-1}}{\langle A u, u \rangle } + \left( \frac{\langle A G^ {-1} A u, u \rangle }{\langle A u, u \rangle } + 1 \right) \frac{u u^*}{\langle A u, u \rangle } \right] , \end{array} \end{aligned}$$

and

67 $$\begin{aligned} \mathrm{Det}(G_+^{-1}, G) = \tau \frac{\langle A u, u \rangle }{\langle A G^{-1} A u, u \rangle } + (1 - \tau ) \frac{\langle G u, u \rangle }{\langle A u, u \rangle }.\end{aligned}$$

### Proof

Denote $\phi :=\phi _{\tau }(A, G, u)$. According to Lemma 6.2 in [27], we have

$$\begin{aligned}&\mathrm{Det}(G^{-1}, G_+) = \phi \frac{\langle A G^{-1} A u, u \rangle }{\langle A u, u \rangle } + (1 - \phi ) \frac{\langle A u, u \rangle }{\langle G u, u \rangle } \;\\&\quad {\mathop {=}\limits ^{(3)}}\; \left[ \tau \frac{\langle A u, u \rangle }{\langle A G^{-1} A u, u \rangle } + (1 - \tau ) \frac{\langle G u, u \rangle }{\langle A u, u \rangle } \right] ^{-1}. \end{aligned}$$

This proves (67) since $\mathrm{Det}(G_+^{-1}, G) = \frac{1}{\mathrm{Det}(G^{-1}, G_+)}$. Let us prove (66). Denote

68 $$\begin{aligned} G_0 :=G - \frac{G u u^* G}{\langle G u, u \rangle } + \frac{A u u^* A}{\langle A u, u \rangle }, \qquad s :=\frac{A u}{\langle A u, u \rangle } - \frac{G u}{\langle G u, u \rangle }. \end{aligned}$$

Note that

69 $$\begin{aligned} G_+&{\mathop {=}\limits ^{(2)}} G_0 + \phi \left[ \frac{\langle G u, u \rangle A u u^* A}{\langle A u, u \rangle ^2} + \frac{G u u^* G}{\langle G u, u \rangle } - \frac{\langle A u u^* G + G u u^* A}{\langle A u, u \rangle } \right] \nonumber \\&\quad \;=\; G_0 + \phi \langle G u, u \rangle s s^*. \end{aligned}$$

Let $I_{\mathbb {E}}$ and $I_{\mathbb {E}^*}$ be the identity operators in $\mathbb {E}$ and $\mathbb {E}^*$. Since $G_0 u = A u$, we have

$$\begin{aligned} \begin{array}{rcl} &{}&{} \left[ \left( I_{\mathbb {E}} - \frac{u u^* A}{\langle A u, u \rangle } \right) G^{-1} \left( I_{\mathbb {E}^*} - \frac{A u u^*}{\langle A u, u \rangle } \right) + \frac{u u^*}{\langle A u, u \rangle } \right] G_0 \\ &{}=&{} \left( I_{\mathbb {E}} - \frac{u u^* A}{\langle A u, u \rangle } \right) G^{-1} \left( G_0 - \frac{A u u^* A}{\langle A u, u \rangle } \right) + \frac{u u^* A}{\langle A u, u \rangle } \\ &{}{\mathop {=}\limits ^{(68)}}&{} \left( I_{\mathbb {E}} - \frac{u u^* A}{\langle A u, u \rangle } \right) G^{-1} \left( G - \frac{G u u^* G}{\langle G u, u \rangle } \right) + \frac{u u^* A}{\langle A u, u \rangle } \;=\; I_{\mathbb {E}}. \end{array} \end{aligned}$$

Hence, we can conclude that

$$\begin{aligned} \begin{array}{rcl} G_0^{-1} &{}=&{} \left( I_{\mathbb {E}} - \frac{u u^* A}{\langle A u, u \rangle } \right) G^{-1} \left( I_{\mathbb {E}^*} - \frac{A u u^*}{\langle A u, u \rangle } \right) + \frac{u u^*}{\langle A u, u \rangle } \\ &{}=&{} G^{-1} - \frac{G^{-1} A u u^* + u u^* A G^{-1}}{\langle A u, u \rangle } + \left( \frac{\langle A G^{-1} A u, u \rangle }{\langle A u, u \rangle } + 1 \right) \frac{u u^*}{\langle A u, u \rangle }. \end{array} \end{aligned}$$

Thus, we see that the right-hand side of (66) equals

70 $$\begin{aligned} H_+:= & {} G_0^{-1} - \tau \left[ \frac{\langle A G^{-1} A u, u \rangle u u^*}{\langle A u, u \rangle ^2} + \frac{G^{-1} A u u^* A G^{-1}}{\langle A G^{-1} A u, u \rangle } - \frac{G^{-1} A u u^* + u u^* A G^{-1}}{\langle A u, u \rangle } \right] \nonumber \\= & {} G_0^{-1} - \tau \langle A G^{-1} A u, u \rangle w w^*, \end{aligned}$$

where

71 $$\begin{aligned} w :=\frac{G^{-1} A u}{\langle A G^{-1} A u, u \rangle } - \frac{u}{\langle A u, u \rangle }. \end{aligned}$$

It remains to verify that $H_+ G_+ = I_{\mathbb {E}}$. Clearly,

72 $$\begin{aligned} \begin{array}{rcl} \langle A G^{-1} A u, u \rangle G_0 w &{}{\mathop {=}\limits ^{(71)}}&{} G_0 G^{-1} A u - \frac{\langle A G^{-1} A u, u \rangle G_0 u}{\langle A u, u \rangle } \\ &{}{\mathop {=}\limits ^{(68)}}&{} A u - \frac{\langle A u, u \rangle G u}{\langle G u, u \rangle } \;{\mathop {=}\limits ^{(68)}}\; \langle A u, u \rangle s. \end{array} \end{aligned}$$

Hence,

73 $$\begin{aligned} \begin{array}{rcl} &{}&{}\langle A G^{-1} A u, u \rangle \langle G_0 w, w \rangle \;{\mathop {=}\limits ^{(72)}}\; \langle A u, u \rangle \langle s, w \rangle \;{\mathop {=}\limits ^{(71)}}\; \frac{\langle A u, u \rangle \langle s, G^{-1} A u \rangle }{\langle A G^{-1} A u, u \rangle } - \langle s, u \rangle \\ &{}&{} \qquad \;{\mathop {=}\limits ^{(68)}}\; \frac{\langle A u, u \rangle }{\langle A G^{-1} A u, u \rangle } \left( \frac{\langle A G^{-1} A u, u \rangle }{\langle A u, u \rangle } - \frac{\langle A u, u \rangle }{\langle G u, u \rangle } \right) \;=\; 1 - \frac{\langle A u, u \rangle ^2}{\langle A G^{-1} A u, u \rangle \langle G u, u \rangle }. \end{array} \end{aligned}$$

Consequently,

74 $$\begin{aligned} \begin{array}{rcl} \frac{\langle G u, u \rangle }{\langle A u, u \rangle } H_+ G_0 w w^* G_0 &{}{\mathop {=}\limits ^{(70)}}&{} \frac{\langle G u, u \rangle }{\langle A u, u \rangle } (G_0^ {-1} - \tau \langle A G^{-1} A u, u \rangle w w^*) G_0 w w^* G_0 \\ &{}=&{} \frac{\langle G u, u \rangle }{\langle A u, u \rangle } (1 - \tau \langle A G^{-1} A u, u \rangle \langle G_0 w, w \rangle ) w w^* G_0 \\ &{}{\mathop {=}\limits ^{(73)}}&{} \frac{\langle G u, u \rangle }{\langle A u, u \rangle } \left( 1 - \tau + \tau \frac{\langle A u, u \rangle ^2}{\langle A G^{-1} A u, u \rangle \langle G u, u \rangle } \right) w w^* G_0 \\ &{}=&{} \left[ \tau \frac{\langle A u, u \rangle }{\langle A G^{-1} A u, u \rangle } + (1 - \tau ) \frac{\langle G u, u \rangle }{\langle A u, u \rangle } \right] w w^* G_0. \end{array} \end{aligned}$$

Thus,

$$\begin{aligned}{}\begin{array}[b]{rcl} H_+ G_+ &{}{\mathop {=}\limits ^{(69)}}&{} H_+ ( G_0 + \phi \langle G u, u \rangle s s^* ) \;{\mathop {=}\limits ^{(72)}}\; H_+ \left( G_0 + \phi \frac{\langle A G^{-1} A u, u \rangle ^2}{\langle A u, u \rangle } \frac{\langle G u, u \rangle }{\langle A u, u \rangle } G_0 w w^* G_0 \right) \\ &{}{\mathop {=}\limits ^{(74)}}&{} H_+ G_0 + \phi \frac{\langle A G^{-1} A u, u \rangle ^2}{\langle A u, u \rangle } \left[ \tau \frac{\langle A u, u \rangle }{\langle A G^{-1} A u, u \rangle } + (1 - \tau ) \frac{\langle G u, u \rangle }{\langle A u, u \rangle } \right] \\ &{}{\mathop {=}\limits ^{(3)}}&{} H_+ G_0 + \tau \langle A G^{-1} A u, u \rangle w w^* G_0 \;{\mathop {=}\limits ^{(70)}}\; I_{\mathbb {E}}. \end{array}\ \ \square \end{aligned}$$

## Footnotes

1 This is obvious when $G - A$ is nondegenerate. The general case follows by continuity.

2 We follow the standard convention that the sum over the empty set is defined as 0, so $\xi _0 = 1$. Similarly, the product over the empty set is defined as 1.

3 Hereinafter, $\lceil t \rceil$ for $t > 0$ denotes the smallest positive integer greater or equal to *t*.

4 We will estimate the second sum using (50). However, recall that, at this moment, (50) is proved only up to the index *k*. This is the reason why we move $\lambda _{k+1}$ into the first sum.

5 $D^3 f_0(x)[h, h, h] = \frac{d^3}{d t^3} f_0(x + t h) \big \vert _{t = 0}$ is the third derivative of *f* along the direction *h*.

6 Indeed, according to Theorem 4.1, we have at least $\lambda _k \le (1 - \frac{\mu }{L})^k \lambda _0$ for all $k \ge 0$.
